# What hinders team innovation performance? Three-way interaction of destructive leadership, intra-team conflict, and organizational diversity

**DOI:** 10.3389/fpsyg.2022.879412

**Published:** 2022-09-29

**Authors:** Suk Bong Choi, Ki Baek Jung, Seung-Wan Kang

**Affiliations:** ^1^College of Global Business, Korea University, Sejong City, South Korea; ^2^College of Business, Gachon University, Seongnam, South Korea

**Keywords:** destructive leadership, intra-team conflict, organizational diversity, innovation performance, three-way interaction

## Abstract

This paper aims to clarify the impact of destructive leadership on team innovation performance. It also explores the relevant conditions that maximize the above relationship. Specifically we examine how intra-team conflict organizational diversity moderate the relationship between destructive leadership team innovation performance. Finally the three-way interaction between destructive leadership intra-team conflict organizational diversity is analyzed for the worst conditions to maximize the negative effect of destructive leadership on team innovation performance. This paper used a cross-sectional design with questionnaires administered to 87 teams with 479 team members working in Korean manufacturing service firms. It applied a hierarchical regression analysis to test the hypothesized relationships including three-way interaction effect among destructive leadership intra-team conflict organizational diversity on team innovation performance. This paper provided empirical insights about how destructive behaviors of team leader hindered team innovation performance. The three-way interaction effects also revealed that the higher the levels of both intra-team conflict organizational diversity the greater the negative effect of destructive leadership on team innovation performance. This paper demonstrates how team leaders’ behavior team organizational conditions result in discouraging overall innovation outcomes. This paper contributes to the innovation leadership literatures by identifying possible leadership type hindering innovation performance at team level the specific conditions their dynamic interaction strengthening the negative effect of destructive leadership on team innovation performance.

## Introduction

Previous studies have analyzed negative leadership through concepts such as abusive supervision (Tepper, 2000; Fischer et al., 2021), bullies (Namie and Namie, 2000), derailed leaders (Shackleton, 1995), psychopaths (Furnham and Taylor, 2004), and toxic leaders (Lipman-Blumen, 2006). Leaders’ destructive behavior, including taking actions not aligned with the goals of the organization, has also been studied (McCall and Lombardo, 1983; Lipman-Blumen, 2006; Einarsen et al., 2007). Based on these studies, destructive leadership can be defined as a repetitive negative behavior by a leader that harms the organization by hindering its goals, resources, and efficiency as well as members’ motivation, well-being, and job satisfaction (Einarsen et al., 2007). This leader’s destructive and abusive behaviors also influenced a psychosocial work environment which in turn has been related to negative health outcomes of employee (Useche et al., 2019). This psychosocial work environment covers matters concerning both our work and all aspect of our working conditions including leaders’ behavior, leader-member interaction, and conflict and diversity situation among work team members. Thus, understanding effects of negative leadership style and work condition is an important clue for reducing employee stress and burnout, and eventually increasing their health and welfare (Sangal et al., 2021). In this vein, destructive leadership makes employees experience a negative work environment. These negative work environments will negatively affect the well-being and health of employees by increasing the workload and workaholism (Molino et al., 2019), sleep problems (Salanova et al., 2016). It also negatively affect psychological recovery, resulting in burnout and exhaustion (Derks and Bakker, 2014; Molino et al., 2019), and unnecessary intra-conflict situation (Jehn et al., 2008; Leon-Perez et al., 2016; Tafvelin et al., 2020).

Destructive leadership which is our main topic has the following characteristics. First, it includes various behavior types of destructive leaders such as physical, verbal, active, passive, direct, and indirect behaviors that hinder employees’ work motivation (Namie and Namie, 2000; Tepper, 2000). Second, a leader may misuse or abuse their power to harm the organization and its members. Their destructive behavior not only reduces member motivation and satisfaction, but also weakens organizational efficiency (Vredenburgh and Brender, 1998). We speculate that it profoundly influences employees’ innovative intention and eventually team innovation performance. Third, destructive leadership is systematic and repetitive, for example, behavior that is repeated every week, or over 6 months (Einarsen et al., 2003). Fourth, the leader’s intent is not a factor for consideration. Although they may have no intention of causing harm, their destructive behaviors can result from carelessness, insensitivity, or lack of leadership capability (Einarsen et al., 2007). Fifth, destructive leadership includes the violation of the legitimate interest of the organization which according to Sackett and DeVore (2001) is illegal, immoral, or deviant. For example, behavior that goes against the legitimate decisions, goals, or strategies of an organization can be regarded as destructive behavior (Einarsen et al., 2007). Additionally, national and international standards, laws, and contracts must be implemented by all members of the organization and infringing these regulations can be considered destructive leadership (Einarsen et al., 2007). Destructive leadership is being actively studied in the organizational behavior area. For example, Wu et al. (2018) addressed the mechanism of destructive leadership and found that employees may show to silence due to their feeling of role conflict, role ambiguity, and role overload when they suffered destructive leadership. Furthermore, a high level of job complexity made the negative effect of destructive leadership even worse. Moreover, other study (Molino et al., 2019) found a positive relationship between destructive leadership and workload, off-work hour technology-assisted job demand (off-TAJD), and workaholism. In addition, they found that both workload and workaholism mediated the relationship between destructive leadership and exhaustion. According to Dolce et al. (2020), destructive leadership was related to exhaustion *via* autonomy. Moreover, they found that autonomy, cognitive demands, and off-work-hours technology-assisted job demand mediated the relationship between destructive leadership and recovery.

Innovation refers to the successful implementation of new ideas by members (Zhou and George, 2001), as well as the introduction of new products and services (Klingebiel and Rammer, 2014). Innovation performance is a part of business performance and makes a significant contribution to the survival and prosperity of the company. Previous studies have identified knowledge sharing (Hu et al., 2009), transformative leadership (Pieterse et al., 2010), shared leadership (Cox et al., 2003), creative self-efficiency (Newman et al., 2018), voice behavior (Guzman and Espejo, 2019), and openness and knowledge (Wang et al., 2020) as antecedents that positively influence and increase a company’s innovation. However, we believe destructive leadership negatively impacts team innovation performance based on various negative effects such as increased stress from the effected company performance, job satisfaction, positive self-evaluation, and well-being, exhaustion (Schyns and Schilling, 2013; Dolce et al., 2020).

Majority of previous studies on innovation have focused on positive factors increasing innovation at individual and organizational levels (Anderson et al., 2004; Crescenzi and Gagliardi, 2018; Newman et al., 2018; Ali et al., 2020; Kaya et al., 2020). These studies have discovered positive effects of leadership types with other positive antecedents of innovation. As leadership is seen as an important driver of innovation, many studies have actively conducted the effects of positive leader’s behaviors that increase creativity and innovative performance (Hughes et al., 2018; Ali et al., 2020). However, few studies have analyzed factors that hinder innovation, especially at the team level. Additionally, some studies have found that leaders do not show positive behaviors in stressful or crisis situations that ultimately lead to negative outcome (Brandebo, 2020). Hence, this study examined negative leadership styles and contexts that hinder team innovation performance. Destructive leadership refers to the leader’s systematic and repetitive behavior that negatively affects the achievement of organizational goals and exacerbates the team members’ motivation (Einarsen et al., 2007). Such destructive leadership continuously abuses the members of the team and negatively impacts their job satisfaction, motivation, and behavior (Schyns and Schilling, 2013; Brandebo, 2020; Mackey et al., 2021), and innovative behaviors, creativity and ultimately reduces innovation performance (Lee et al., 2020).

This study investigated the conditions under which destructive leadership hinders innovation and analyses factors that magnify or weaken these conditions. By examining the direct effects of destructive leadership on team innovation performance, we seek conditions moderating this relationship. In this sense, we bring together two important moderating factors from team and organizational perspectives. First, we postulate that the level of intra-team conflict has a negative impact on destructive leadership and innovation performance by increasing conflict and disagreement between members of the team (De Dreu and Weingart, 2003). In other words, if the level of intra-team conflict is high due to interaction between team members, the team leader’s destructive leadership is expected to further reduce team innovation performance (Badke-Schaub et al., 2010). Second, this study analyzed the effect of organizational diversity as an organizational factor that affects team innovation performance. Organizational diversity refers to the degree of difference among team members in age and gender, as well as their different opinions, knowledge, skills, values, and principles (Dahlin et al., 2005). Recent studies have already revealed that organizational diversity provides various ideas or methods for problems that arise while working (Jehn et al., 1999; Horwitz and Horwitz, 2007). Conversely, it may also lead to conflicts while implementing organizational strategy and delays the decision-making process (Shemla et al., 2016; Chen et al., 2019). Therefore, we expected that the negative effects of destructive leadership on team innovation performance will worsen by level of organizational diversity. Furthermore, we analyzed the three-way interaction effects of individual and organizational variables such as destructive leadership, intra-team conflict, and organizational diversity. Thus, the detailed and dynamic conditions that maximize negative effects of destructive leadership on innovation performance were identified through an integrated approach.

## Theoretical background and research hypotheses

Employees’ attitude and behavior toward organization can be influenced by many factors which organizations have various contexts. Thus, the use of one theoretical lens is not sufficient to understand the organizational performance. For this reason, we used several theories for building the hypotheses of this study.

First, leader behavior strongly affects the organizational member’s attitude and behavior. Social exchange theory explains social interaction through the economic and social exchange process (Blau, 1964). Blau (1964) suggested trust as an important antecedent for establishing better relationships because social exchange relationships have voluntary and informal characteristics. According to the social exchange theory (Blau, 1964), organizational members who have received favorable treatment and support from the organization and leaders, have a sense of obligation to effort for the organization. In this vein, employees who received abusive supervision and unfair treatment by leader will have negative sense and emotion toward leader and organization. Therefore, employees’ trust, intrinsic motivation, and obligation toward the team innovation will be decreased. As a result, we assume that employees who experienced the leaders’ destructive behavior have negative perception and behaviors on team innovation activity **(Hypothesis 1).**

Second, threat-rigidity theory suggested that members, teams, and organizations exhibit rigidity or inability to act and do something new, in the face of adversity (Staw et al., 1981). Hence, it suggested that perceived social threat, as in relationship conflict, activates a stress reaction, which creates cognitive rigidity, defensiveness, closed-mindedness, and avoidance response (Carnevale and Probst, 1998; O’Neill and Mclarnon, 2018). This point is that conflicts among team members negatively affect team innovation because team members cannot make flexible thinking and new attempts. Thus, we can assume that in leaders with high destructive leadership, teams with higher conflict would have a higher pain among team members thereby decreasing team innovation performance (**Hypothesis 2).**

Third, we also apply the social categorization theory (Tajfel, 1981) to address that organizational diversity moderates the relationship between destructive leadership and team innovation performance. Social categorization theory (Tajfel, 1981) explain that people do not feel prejudiced or attracted to heterogeneous groups because they categorize themselves by characteristics and those who do not. Similarly, people are attracted to others with similar characteristics (Byrne, 1971). Referring to the premises of the this theory, organizational members can be further categorized into smaller groups with regard to diversity elements such as age, gender, education, and experience (Telyani et al., 2022). Segmentation within a team based on a variety of factors can further reinforce the negative effects of disruptive leadership by increasing the negative attitudes and behaviors of team innovation by team members who have experienced disruptive leadership behavior (**Hypothesis 3).**

Finally, contingency theory postulates that no one best management method exists (Donaldson, 2001). Therefore, theorists who argued for contingency theory tried to identify the meet point of the organization and environment for high performance. Based on contingency theory, prior studies investigated contingent factors that could activate the negative or positive side of diversity (Van Knippenberg et al., 2004; Richard et al., 2007; Wegge et al., 2008; Sung and Choi, 2021a). In this study, we highlighted the role of intra-team conflict and organizational diversity as important contingency factors that enhances or minimizes the effect of destructive leadership on performance (Joshi and Roh, 2009). If organizational diversity is high, the team split a group into subgroups. This leads to conflict and hinders team cohesion and commitment (Zouaghi et al., 2020). Therefore, we expect that both organizational diversity and intra-team conflict which are important contingent factors moderate the relationship between destructive leadership and team innovation performance (**Hypothesis 4).**

### Destructive leadership and team innovation performance

Destructive leadership offends members’ work life in the organization by making unreasonable demands or a mockery of them (Schyns and Schilling, 2013). Destructive leadership increases negative emotions, feeling and attitudes among team members (Tepper et al., 2004; Erickson et al., 2015), causing stress and degrading their well-being. In such situations, the members’ job dedication and satisfaction decrease, causing them to put in less effort, thereby negatively affecting individual innovation performance and eventually reducing team innovation performance. The continuous destructive behavior of the leader makes it difficult for members to maintain long-term motivation for innovation (Aryee et al., 2007; Schyns and Schilling, 2013). Innovative actions based on job satisfaction cannot be expected from members who are less satisfied, committed, and motivated because of destructive leadership (Connolly and Viswesvaran, 2000).

Furthermore, members treated unfairly by destructive leaders feel threatened by interpersonal injustice (Skarlicki and Folger, 1997; Collins and Jackson, 2015), emotional exhaustion (Xu et al., 2015), and identity (Aquino and Douglas, 2003), while also feeling anger and frustration toward the team and the leader. Consequently, undesirable attitudes (Tepper, 2000; Duffy et al., 2002), such as reduced organizational commitment, increased turnover intention (Ashforth, 1997; Iqbal et al., 2021), and increased unproductive work activity (Mitchell and Ambrose, 2007) may be triggered, leading to bad team innovation performance. Therefore, members who are tired of destructive leaders become more focused on controlling their emotions and finding their identity rather than trying to contribute to organizational goals. As a result, insufficient resources and efforts necessary to achieve innovation are put in, which negatively affect team innovation performance.

Members who have suffered great stress and emotional exhaustion from destructive leadership are evasive and passive to ease their psychological distress (Tepper et al., 2007). According to the conservation of resources theory, members adopt strategies to deliberately avoid contact or feedback to minimize resource loss in stressful situations (Hobfoll, 1989; Tepper et al., 2007; Whitman et al., 2014), which negatively influences team innovation performance. As a form of evasive and passive behavior, members engage in passive actions, including organizational silence, reducing work-related productive remarks, not reporting problems, and not proposing new ideas (Xu et al., 2015). That is, members tend to remain silent if the leader shows no interest in their suggestions to solve work-related problems creatively (Vakola and Bouradas, 2005). Constructive and diverse opinions of members in the course of job implementation are an important element for creating new ideas for innovative change and continuous improvement (Van Dyne and LePine, 1998; Ng and Feldman, 2012). However, destructive leadership that leads to the silence of members encourages intentional omission and neglect and eventually negatively affects team innovation performance.

**Hypothesis 1 (H1).** Destructive leadership will have a negative influence on team innovation performance.

### The moderating effect of intra-team conflict

Intra-team conflict refers to a state in which opinions, arguments, and interests clash between two or more team members on an issue (Wall and Callister, 1995; Shetach, 2009). The concept of conflict can be divided into task and relationship conflict. Task conflict refers to a state of conflict in which active discussion and personal excitement among members arise related to the task given by team (Pelled et al., 1999; Simons and Peterson, 2000); continuous task conflicts may lead to negative emotions about interpersonal relationships. Conversely, relationship conflict is the recognition of inconsistency and differences over interpersonal preferences, values, and personality differences, and involves negative emotions (Jehn, 1995; Jehn and Bendersky, 2003). Previous studies have shown that normal task conflict has a positive impact on performance, while relationship conflict has a negative impact (Jehn and Bendersky, 2003; Hu et al., 2019; Downes et al., 2021). De Dreu and Weingart (2003) show that task conflicts have a positive relationship with team performance only partly, with an overall negative relationship with everything else. Thus, the impact of task conflict on organizational performance remains controversial and leads to mixed results (Arazy et al., 2011; Chang, 2020). Therefore, conflict within a team is subject to management, and relationship conflict in particular negatively affects team performance (Badke-Schaub et al., 2010).

Job autonomy, peer support, leader-member exchange relationships, and organizational support awareness have been addressed as organizational environmental factors that promote innovation (Scott and Bruce, 1994; Axtell et al., 2000; Janssen, 2005; Ramamoorthy et al., 2005). Previous studies show that innovative actions by employees were positively moderated by organizational support and relationships with coworkers (Kwon and Kim, 2020). However, if the relationship between team members is not good and conflict occurs, members risk losing motivation for innovation. Intra-team conflict is also a phenomenon caused by interaction between members, and the satisfaction or performance of members depends on the degree of conflict management by the organization (Jehn, 1995; De Dreu and Weingart, 2003; Jehn and Bendersky, 2003; O’Neill et al., 2013; Yin et al., 2020). However, destructive leaders negatively affect members’ psychological condition by continuously abusing them (Park et al., 2018; Ogunfowora et al., 2021). As a result, not only is the members’ immersion in their work reduced, but conflict between members cannot be resolved smoothly, eventually maximizing the negative effects of interaction of destructive leadership and team intra-conflicts.

Conflicts cause lack of communication among members and increase stress, incurring psychological unsafe in the course of completing their task (Suifan et al., 2019; Esbati and Korunka, 2021). Additionally, members become increasingly passive toward implementing given work and creative problem-solving (Nightingale, 1974; De Dreu and Weingart, 2003; Beitler et al., 2018). As previous studies have identified, relationship conflicts increased tension among team members, further hindering their innovation behaviors (Lu et al., 2011). Thus, we assumed that intra-team relationship conflicts which act as a psychological mechanism, causing emotional confrontation and abuse, profoundly hinder knowledge sharing and cooperation among members (De Dreu, 2006; Lu et al., 2011; Vinarski-Peretz et al., 2011). In other words, the higher the intra-team conflict level, the more negatively it will affect the team innovation performance. Therefore, the intra-team conflict level can be expected to further increase the negative effect of destructive leadership on team innovation performance. Based on the above discussion, we established the following hypothesis.

**Hypothesis 2 (H2).** Intra-team conflict level will further strengthen the negative relationship between destructive leadership and team innovation performance. That is to say, the negative impact of destructive leadership on team innovation performance will be further strengthened if the intra-team conflict level is higher than when it is lower.

### The moderating effect of organizational diversity

In the field of organizational behavior, diversity can be explained by differences in a variety of areas (Griggs, 1995) including race (Zopiatis et al., 2014), gender (Pinar et al., 2011), income and education level (Hsiao et al., 2015), length of employment, rank (Kim et al., 2009; Waight and Madera, 2011), and opinions, beliefs, and values (Dahlin et al., 2005). Many studies on organizational diversity have confirmed that it has both positive and negative effects on performance (Soni, 2000; De Meuse and Hostager, 2001; Reynolds et al., 2014). However, according to social identity theory (Tajfel, 1978; Cannella et al., 2015) and self-categorization theory (Turner and Reynolds, 2011), individuals are motivated to enhance their social identity and thus classify their team members into individuals who have the same characteristics as them and those who do not. They show favorable and positive attitudes toward those who have similar characteristics (Tajfel and Turner, 1986; Turner and Haslam, 2001) and a false bias develops between members. Furthermore, according to the similarity-attraction paradigm (Barsade et al., 2000; Hoppe et al., 2014), there is a stronger bond between people who show similarity in interpersonal interaction. Based on these logics, we speculate that the effect of organizational diversity will further amplify the negative effect of destructive leadership on team innovation performance.

In heterogeneous groups with high organizational diversity, members respond positively to those with similar characteristics, while perceiving those who are not similar as less trustworthy and uncooperative (Turner and Reynolds, 2011). This can cause conflict among members and reduce cooperative attitude for innovation (Mohammed and Angell, 2004; Zouaghi et al., 2020). Low level of team cohesion and frequent conflict among members due to high diversity may hinder the innovation process in various ways. For example, organizational diversity increases the differences in characteristics among team members, leading to the level of members’ satisfaction, cooperation, and communication in the course of completing team tasks (Kim and Song, 2020). This also increases the rate of turnovers and conflict and ultimately negatively effects task progress and internal motivation for innovation (Williams and O’Reilly, 1998; Shemla et al., 2016; Homan et al., 2020).

Therefore, if the level of diversity in a team is high, members will not communication actively and they will not focus on achieving the organizational objectives led by the leader. Due to the effect of social categorization, teams with high organizational diversity have low cooperation among members and have a negative effect on the formation of cooperation-oriented norms within the team (Chatman and Flynn, 2001). These again reduce negative effects, undermining team efficiency and effectiveness, increasing the negative effects of disruptive leadership on team process activation. Additionally, by hindering teamwork and making it difficult to share information and knowledge among members (Ancona and Caldwell, 1992; Kim, 2018), organizational diversity contributes to further strengthening the hindering effect of a team leader’s destructive leadership on innovation performance. Furthermore, organizational diversity prevents them from overcoming the negative effects of destructive leadership (Smith et al., 1994). Destructive leaders often engage in destructive behavior due to their lack of capability in leading members, rather than pursuing innovation to achieve organizational goals more effectively (Einarsen et al., 2007). Diversity, when managed, is also a source of creativity and innovation, but the behavior of destructive leaders strengthens the short of diversity management. Therefore, diversity management will not be able to reduce the negative effects of diversity experienced by members. Furthermore, organizational diversity will amplify the negative impact on innovation by interacting with destructive leadership. Based on the above discussion, we established the following hypothesis.

**Hypothesis 3 (H3).** Organizational diversity will further strengthen the negative relationship between destructive leadership and team innovation performance. That is to say, the negative impact of destructive leadership on team innovation performance will be further strengthened if the level of organizational diversity is higher than when it is lower.

### Integrated model: Three-way interaction

Based on the above discussions, we hypothesized that intra-team conflict and organizational diversity mutually interact and negatively influence team innovation performance and that they interact with destructive leadership and further reduce team innovation performance. In other words, we anticipated that the organizational diversity interacts with destructive leadership and intra-team conflict negatively influences team innovation performance. As mentioned earlier, in relation to the moderating effect of organizational diversity, a team with a high level of organizational diversity interacts with destructive leadership and escalates the negative effect of intra-team conflict on team innovation performance by decreasing team cohesion and delaying decision-making on implementation of innovative problem-solving trial.

Specifically, intra-team conflict not only weakens trust among members, but also reduces organizational commitment, bringing about a negative attitude in members toward the organization and their work. Therefore, the negative relationship between destructive leadership and team innovation performance will change depending on the intra-team conflict level. Furthermore, if organizational diversity at the team level is high as well, the team’s cohesion and trust will be weakened, further strengthening the negative relationship between destructive leadership and team innovation performance. In other words, the higher the organizational diversity, the more the negative effects of intra-team conflict and destructive leadership. Similarly, if organizational diversity exists within the team, members go through the social classification process and become biased, thereby weakening team cohesion, collaboration, and eventually innovation (Williams and O’Reilly, 1998; Shemla et al., 2016; Kim, 2018). Furthermore, if there is a high level of conflict among team members, such negative effects of organizational diversity will be even greater. In other words, if the level of intra-team conflict and organizational diversity are simultaneously high, members will not be able to cooperate and the negative effects of destructive leadership (innovation performance) will increase due to the stress caused by conflict and diversity. Therefore, the following hypothesis was established.

**Hypothesis 4 (H4).** The intra-team conflict and organizational diversity will have a moderating effect on the relationship between destructive leadership and team innovation performance. That is to say, when both the intra-team conflict level and organizational diversity are high, the relationship between destructive leadership and team innovation performance will become more negative.

**FIGURE 1:**
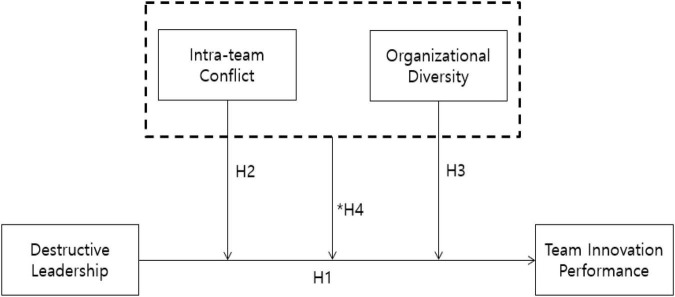
Hypothesized research model. *Three-way interaction (H4).

The hypothesized research model is presented in Figure 1.

## Methodology

### Data collection and sample characteristics

To conduct an empirical analysis, we collected data from Korean firms. Data collection for this study was conducted through an online and offline questionnaire. To carry out the online and offline survey procedure, we collected the contact information of each team leader in advance. We tried to ensure anonymity in the course of survey. In the first step, we contacted the team leader to explain the purpose of the survey. In the second step, when we obtained their permission, we requested to ensure anonymity and consider the diversity of samplings such as gender, tenure, and age. Next, we distributed anonymous online and offline questionnaires to employees. Participants were explained through the purpose and procedures of the survey and the benefits and disadvantages that may arise from participating. An informed consent was obtained from all participants involved in the study. Moreover, we guided them to have the freedom to withdraw from the survey at any time. Based on their acceptance, we collected data from 95 teams (460 team members), of which 87 teams (429 team members) provided valid responses that were used for this study. The proper sample size in this research has followed that of previous studies published in main journals of this filed. The sample size was determined by referring to prior studies which are similar to the subject of this study such as team-level study (Kipkosgei et al., 2020), team innovation (Liang et al., 2019), leadership and team innovation (Tang et al., 2020), innovative behavior (Choi et al., 2016; Saeed et al., 2018), and creativity study (Zhang and Bartol, 2010). Thus, we confirmed that the number of samples used in this study was proper for empirical analysis. The demographic characteristics of the respondents are as follows. Each team comprised a minimum of four and a maximum of nine members. The average size of the team was 4.93 (SD = 1.45). Among the 429 respondents, 91.4% were male and 8.6% were female. The average age of the respondents was 40.32 (SD = 8.06) and average tenure was 13.66 (SD = 8.59).

### Measures

The questionnaire used in this study was originally prepared in English and translated into Korean. We followed Brislin’s (1980) back translation procedure. A professional translator translated the original version into Korean, which was then back-translated into English by a bilingual scholar who had no prior knowledge of the objectives of the study and had not seen the original survey.

#### Destructive leadership

Destructive leadership is defined as leadership in which the leader continuously manages the members in an abusive supervision. Leadership was measured by employees’ perceptions of their team leader behavior. In this study, leaders refer to those who have the authority to evaluate employees’ performance such as department head, team manager, and supervisors. We used the five items that were used by Mitchell and Ambrose (2007) (see Appendix A). This measurement was based on Tepper’s (2000) abusive supervision measures. Mitchell and Ambrose (2007) further developed by focusing on active interpersonal abuse by the supervisor (e.g., “ridicules me” and “tells me my thoughts and feelings are stupid”). Because the active dimension is more consistent with our research interest, we used this Mitchell and Ambrose (2007)’ 5 items version as our indicator of destructive leadership. Sample items include the following: “My team leader ridicules me.” and “My team leader puts me down in front of others.” The Cronbach’s alpha was 0.879.

#### Intra-team conflict

Intra-team conflict is defined as relational conflict including negative emotions, recognizing inconsistencies and confrontations on interpersonal preferences, values, and differences in personality outside of work (Jehn, 1995). We used the four items that were developed by Jehn (1995). Sample items include: “Our team tends to have personality conflicts between members” and “Our team tends to have emotional conflicts between members.” The Cronbach’s alpha was 0.904.

#### Organizational diversity

Organizational diversity refers to the diversity of members’ gender, age, education level, and tenure. The combined measure of diversity was created by calculating prior study (Schippers et al., 2003). Standard deviation was used for the age and tenure of the members (Bedeian and Mossholder, 2000; Harrison et al., 2002) and gender was used as the proportion of minority members in the team (Kanter, 1977; Pelled, 1996). Education level was calculated according to the calculation method of Blau’s index (Blau, 1977). Organizational diversity index ranged also from 0 to 1, where a higher score indicated a greater distribution of demographic characteristics within the team, thus indicating higher levels of diversity. For the sample, organizational diversity ranged from 0.01 to 0.64 (*M* = 0.28, SD = 0.13).

#### Team innovation performance

Team innovation performance is defined as the level of effort by which team members perform work efficiently to achieve goals and to practice innovative actions (Pirola-Merlo and Mann, 2004). We used the four items developed by Pirola-Merlo and Mann (2004). Sample items include the following: “My team’s recent output is new” and “My team’s recent output is creative.” We aggregated the responses of members to use individual-level variables as team-level group variables. The Cronbach’s alpha was 0.887.

#### Control variables

The control variables included the team members’ average marital status, wage, job type and position, and team size, which could affect innovation performance (Katz, 1982; Ancona and Caldwell, 1992; Brown and Eisenhard, 1995). The control variables were used at the team level.

## Results

### Preliminary analysis

Since all data were collected at the same source, it could have resulted in the common method bias (CMB), leading to false internal consistency and potentially misleading results. Thus, to assess the effect of common method variance, we followed the recommendation by Podsakoff et al. (2003) and conducted Harman’s single-factor test by loading all the items of the study constructs into an exploratory factor analysis. The results indicated that no single factor explained more than 28% of the covariance among the variables. Thus, it was concluded that CMB did not significantly alter the validity of the study results.

To analyze the respondents’ individual-level data at the team level, the within-group agreement rWG(J) index (James et al., 1993) was used to aggregate responses. To further analyze variation in the data, individual responses were matched to team membership by calculating interclass correlation coefficients (ICC): ICC (1) and (2). Then, the hypotheses were tested using hierarchical regression analysis. A rWG(J) index value larger than 0.70 is considered to represent a satisfactory agreement within a group (Bliese, 2000). ICC (1) was used to measure the inter-respondent reliability, with a range of 0.05–0.30 or statistical significance considered adequate. ICC (2) assessed the mean reliability of a group; previous literature suggested that values of 0.70 or larger are acceptable and values between 0.50 and 0.70 are marginally acceptable (Kipkosgei et al., 2020). Table 1 presents the rWG(J), ICC (1) and (2) results. All values were above the acceptable standard cut-off values, indicating that all values were within the acceptable range.

**TABLE 1:** rWG(J), ICC (1), ICC (2) of all team-level variables.

Variables	rWG (J)	ICC (1)	ICC (2)
Destructive Leadership	0.91	0.23	0.59
Team Innovation Performance	0.92	0.17	0.51
Intra-team Conflict	0.87	0.26	0.63

### Correlation and reliability analyses

We conducted a correlation analysis to investigate the relationship between the measured variables and their direction. The means, standard deviations, reliabilities, and correlations among the key variables are shown in Table 2. As can be seen, there was a correlation (*p* < 0.001) between destructive leadership, intra-team conflict, and team innovation performance. Team-level variables are mean centered to solve the multicollinearity problem (Aiken et al., 1991). The maximum variance inflated index of the key variables is 3.11. Hence, there was no multicollinearity problem (Hocking and Pendleton, 1983).

**TABLE 2:** Means, standard deviations, correlations, and reliabilities.

Variables	M	SD	1	2	3	4	5	6	7	8	9
1. Team size	4.93	1.45	–								
2. Marital status	1.72	0.22	0.020	–							
3. Wage	3.56	0.66	–0.133	0.580***	–						
4. Job type	1.93	0.92	0.060	0.158	–0.128	–					
5. Position	4.08	0.81	−0.211*	0.509***	0.792***	−0.217*	–				
6. DL	1.58	0.39	−0.242*	0.078	0.032	0.035	0.053	(0.879)			
7. IC	2.14	0.51	–0.079	0.186	0.281**	0.035	0.172	0.472***	(0.904)		
8. OD	0.28	0.13	0.057	−0.591***	−0.598***	0.155	−0.546***	0.085	–0.009	–	
9. IP	3.29	0.38	0.123	–0.009	–0.084	0.087	–0.056	−0.517***	−0.449***	0.032	(0.877)

*N* = 87. *p < 0.05; **p < 0.01; ***p < 0.001, Cronbach’s alpha coefficients are reported in diagonal; Destructive Leadership (DL); Intra-team Conflict (IC); Organizational Diversity (OD); Team Innovation Performance (IP).

### Hypothesis testing

We used hierarchical regression analysis to test the hypothesized relationships (Wiklund and Shepherd, 2005; Hoch et al., 2010). The results of the hierarchical regression analysis are shown in Table 3. Hypothesis 1 stated that destructive leadership would have a negative effect on team innovation performance. In Model 2, destructive leadership was found to negatively affect team innovation performance (β = −0.529, *p* < 0.001), supporting Hypothesis 1. As shown in Model 4, the interaction term of destructive leadership and intra-team conflict was negatively related to team innovation performance (β = −0.260, *p* < 0.01). Thus, Hypothesis 2 was supported. However, the interaction term of destructive leadership and organizational diversity did not significantly affect team innovation performance (β = −0.097, n.s.). Thus, Hypothesis 3 was not supported. To test Hypothesis 4, we studied the three-way interaction of destructive leadership, intra-team conflict, and organizational diversity for predicting team innovation performance in the last step of the moderated hierarchical regression model. Here, with β = −0.279 (*p* < 0.01), we found a significant negative effect in predicting team innovation performance.

**TABLE 3:** Summary of regression analysis results.

Variables	Team innovation performance				
	Model 1	Model 2	Model 3	Model 4	Model 5
**Step 1: Control variables**					
Team size	0.118	–0.017	–0.006	–0.004	0.030
Marital status	0.009	0.073	0.136	0.161	0.161
Wage	–0.131	–0.165	–0.009	–0.009	0.037
Job TYPE	0.080	0.091	0.076	0.137	0.183
Position	0.085	0.081	0.055	0.085	0.077
**Step 2: Main effect**					
DL		−0.529***	−0.411***	−0.335**	−0.357**
**Step 3: Moderators**					
IC			−0.289*	−0.304**	−0.256*
OD			0.158	0.154	0.237
**Step 4: Two-way interaction**					
DL × IC				−0.260**	−0.290**
DL × OD				–0.097	–0.020
IC × OD				0.215*	0.146
**Step 5: Three-way interaction**					
DL × IC × OD					−0.279**
R^2^	0.028	0.288	0.350	0.438	0.494
R^2^ change		0.260***	0.062*	0.088*	0.056**

*N* = 87; *p < 0.05; **p < 0.01; ***p < 0.001(two-tailed test); Standardized regression coefficients reported; Destructive Leadership (DL); Intra-team Conflict (IC); Organizational Diversity (OD).

**FIGURE 2:**
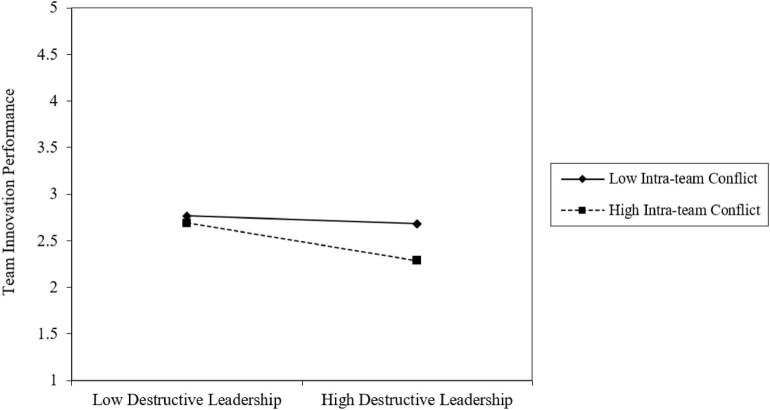
Moderating effect of intra-team conflict on the relationship between destructive leadership and team innovation performance.

**FIGURE 3:**
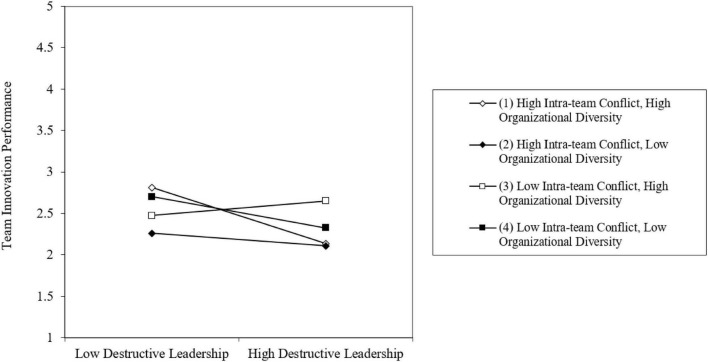
Three-way interaction effect of destructive leadership, intra-team conflict, and organizational diversity on team innovation performance.

As shown in Figure 2, when destructive leadership and intra-team conflict are high, the negative relationship between destructive leadership and team innovation performance is stronger. Moreover, as shown in Figure 3, when destructive leadership, intra-team conflict, and organizational diversity are high, the negative relationship between destructive leadership and team innovation performance is stronger than whey they are low. Thus, Hypothesis 4 was supported.

## Discussion

This study aimed to examine the interaction effects of intra-team conflict and organization diversity on the relationship between destructive leadership and team innovation performance. The core findings of our empirical analysis can be summarized as follows. First, we found that destructive leadership negatively influenced team innovation performance. Second, the negative effect of destructive leadership on team innovation performance was strengthened when intra-team conflict level was high. Third, organizational diversity did not significantly strengthen the relationship between destructive leadership and team innovation performance. Fourth, the relationship between destructive leadership and team innovation performance was more negative when both intra-team conflict and organizational diversity were high. A previous study on leadership with negative effects, such as destructive leadership, show that the destructive behaviors of leaders negatively affect not only innovation performance, but also well-being, job satisfaction, and corporate performance (Schyns and Schilling, 2013). In this context, this study sought to identify the specific conditions or situations in which the negative effect of destructive leadership on team innovation performance is maximized. Our results show that intra-team conflict is a moderating variable that increases the negative relationship between destructive leadership and team innovation performance. However, organizational diversity was shown to not have moderating effects. Analysis using the three-way interaction model shows that the negative effect of destructive leadership on the team innovation performance was found to be strongest when both the intra-team conflict level and organizational diversity are high.

### Theoretical contributions

Based on the empirical analysis of this study, the following theoretical implications were derived. First, previous research on destructive leadership have generally focused on verifying its negative effects but have not been able to draw a clear conclusion about the relationship between destructive leadership and organizational performance (Detert et al., 2007; De Hoogh and Den Hartog, 2008). In this vein, our results also confirm that social exchange theory was worked in explaining the negative relationship between destructive leadership and team innovation performance. Therefore, we have contributed by extending it to leadership study and Korean workers context. Recent research indicates that a more systematic study is needed on the extent of the effect of destructive leadership on individual or team performance (Kellerman, 2004; Kelloway et al., 2005). Therefore, this study focused on exploring factors that amplify the negative effect of destructive leadership, taking into consideration the possibility that the effects of destructive leadership on performance may vary depending on complex internal and external characteristics of the organization. Our results confirm that the negative effects of destructive leadership are strengthened when members are exposed to conflict situations within the team. The results of this study are consistent with the demands of previous studies to focus on the importance of a psychosocial working environment for employee happiness and health creation (Useche et al., 2019). In such a situation, members will not only be negatively affected psychologically by the destructive behavior of the leader, but also lose motivation to work due to conflict with other members which is not handled properly by the leader. Therefore, this study explored situational factors that increase the negative effects of destructive leadership.

Second, organizational diversity, the second moderating variable of this study, was predicted to have a negative effect on team innovation performance through interaction with destructive leadership but showed non-significant results. It can be inferred that the more diverse the members, the less the negative effect of destructive leadership based on the positive interaction between members. This result was also explained through previous studies on diversity that confirmed its positive aspects (Watson et al., 1993; Drach-Zahavy and Somech, 2001). There are several benefits to diversity among team members, such as improved quality in creativity, innovation, and performance (Watson et al., 1993; Drach-Zahavy and Somech, 2001). There is an advantage in achieving innovation in the process of collecting information from different individuals and presenting various solutions (Northcraft et al., 1995; Jehn et al., 1999; Kickul and Gundry, 2001). Furthermore, a highly diverse group pursues innovative strategies and has a positive influence on performance (Richard, 2000). It can challenge past practices and make members more open to change, which can drive organizational flexibility and strategic change (Wiersema and Bantel, 1992; Boeker, 1997; Cummings, 2004; Roberson et al., 2017). Therefore, this study is meaningful in that it examined the effect of diversity, which has both negative and positive effects in the process of destructive leadership affecting team innovation performance.

Third, various situational factors may exist in the process of how destructive leadership negatively impacted team innovation performance. However, there are few studies that have analyzed these conditions by simultaneously applying them, especially at the team level. This study found that the three-way interaction between destructive leadership, intra-team conflict, and organizational diversity, that affect team innovation performance, is effective. It was shown that when intra-team conflict level and organizational diversity are high, destructive leadership highly negatively impacts team innovation performance. What is noteworthy is that when intra-team conflict level is low while organizational diversity is high, team innovation performance increases. It can be inferred that this is because of the positive effects of diversity as suggested by other studies (Watson et al., 1993; Drach-Zahavy and Somech, 2001; Kickul and Gundry, 2001). As such, this study closely analyzed the conditions for maximizing the damage of destructive leadership in a team. As presented by Burke (2006), this study broadened the research on leadership by exploring the “dark side” of leadership which has been underdeveloped. In addition, we confirmed the role of moderating variables to strengthen the relationship between destructive leadership and team innovation performance through threat-rigidity theory, social categorization theory. We also explained the three interaction effects of two moderating variables based on the contingency theory. Our findings confirmed that these theories dealt with in organizational behavior research could be operating in the Korean employee context. Thus, these results contributed to theoretical expansion as they revealed the applicability of existing theories.

Lastly, most of the research on the negative behaviors of leaders has focused on the individual level (McCall and Lombardo, 1983; Lipman-Blumen, 2006; Einarsen et al., 2007). This study is meaningful as it expands individual-level research into team-level research.

### Managerial implications

The practical implications of this study are as follows. First, it identified that destructive leadership is a key factor that hinders team innovation performance. Therefore, organizations should strengthen their leadership education programs so that team leaders can avoid destructive leadership and adopt better leadership styles. Moreover recently, most Korean companies have rapidly applied a performance-oriented and hierarchically operated system to survive in global competition, destructive leadership appears relatively frequently in Korea due to the emergence of supervisors empathizing high pressure for superior performance (Ashkanasy, 2002; Lee et al., 2013). However, work-life balance, employees’ well-being, welfare, and health in workplace are importantly considered as a source of sustainable society in recent South Korea. In this vein, there is a tendency to change a work climate in which employees do not make enough efforts for the performance when they are treated unfairly by leaders and organizations. Thus, we confirmed that destructive leader behaviors prevailed in Korean workplaces to push their employees to meet performance expectations incur more harm than benefit in the South Korean context. Thus, organization should establish a proper control system and culture to reduce the destructive leadership.

Second, considering that intra-team conflict is an important factor that increases the negative effect of destructive leadership, top management must recognize the importance of conflict management for team members. Team members may work in a constructive way through interaction, cooperation, and knowledge sharing but their relationship conflicts may negatively affect these work-related actions (Lu et al., 2011), eventually reducing team innovation performance. When experiencing conflict with other members, the leaders’ conflict management ability will be most important. Therefore, they must pay attention to conflicts between team members and manage them appropriately. Traditionally, Korean culture is characterized by collectivism, high power distance, and uncertainty avoidance (Lee and Lee, 2014). Korean employees are basically sensitive to incurring interpersonal tensions and involving group conflict issues because of collectivism tradition emphasizing the community interest and harmony than individuals’ preference (Sung and Choi, 2021b). The Confucianism prevailed in Korean society have also emphasized harmonious relationships among leader and follower as well as organizational members, so any sort of intra-conflict is less accepted (Lee et al., 2018). In this vein, proper conflict management is the crucial point for enhancing organizational innovative capability and effectiveness of Korean firms.

Third, a direct moderating effect of organizational diversity in the relationship between destructive leadership and team innovation performance could not be found in this study. However, when conflict within the team intensified, negative effects of organizational diversity were found. Therefore, to prevent negative effects and encourage positive effects of organizational diversity, top management must put in additional effort to maintain an appropriate level of organizational diversity.

### Limitations and future research

This study also has a few limitations despite its theoretical and practical contributions. Future researchers should consider our several limitations. First, our variables were collected from a self-reported questionnaire at the same time from the same source. Although, previous study could not find strong evidence that self-reported questions prevent meaningful interpretations of data, self-report data can show common method bias (Chan, 2009). To reduce this problem, we efforted to ensure anonymity in the data collection process. In addition, organizational diversity was measured using gender, age, education level and tenure (Schippers et al., 2003). Despite our efforts, future studies should collect data from various sources and different times. For example, team innovation performance should be measured by different sources like R&D expenditure, number of new product and patent registration at team level.

Second, this study was designed as cross-sectional research, therefore, the causal relationship between destructive leadership and team innovation performance could not be analyzed in depth. Although this study hypothesized that the destructive leadership of the team leader undermines team innovation performance, the effect of destructive leadership on team innovation performance can change over time. Therefore, longitudinal research could be designed in the future to analyze the causal relationship between destructive leadership and team innovation performance more accurately.

Third, our data were collected from South Korean employees only, it is possible that the cultural background significantly influenced employees’ attitudes and perceptions. Therefore, our results had limitations in generalizing the findings to other sectors and firms in different countries. Future researchers may replicate our findings in different country contexts.

Fourth, this study found important roles of moderating variables in maximizing the negative effect of destructive leadership on team innovation performance. However, we suggest that examining the various frames can provide meaningful implications such as mediating relationship. Especially, previous research suggested a curvilinear relationship between abusive supervision and creativity (Lee et al., 2013). Thus, if future studies explore the various paths and process to explain how destructive leadership led to innovation performance, they could contribute to the research on explaining the effects of negative destructive leadership and enhancing team innovation. In addition, as the health of workers in Korean workplaces has recently attracted much attention, useful implications can be obtained if the destructive leadership and intra-conflict issues dealt with in this paper, are expanded to the studies of health and welfare of workers in future.

## Conclusion

In summary, this study investigated the important situational factors that maximize the effect of destructive leadership on team innovation performance. The findings of our study confirmed the negative effect of destructive leadership on team innovation performance. Moreover, we found that intra-team conflict played moderating roles in enhancing the negative effect of destructive leadership. In addition, this study also highlighted the importance of interaction between the three factor- destructive leadership, intra-team conflict and organizational diversity- for team innovation performance. We found something interesting in the effects of the three-way interaction. It was found that the negative impact of destructive leadership was large under three conditions, excluding low intra-team conflict and high organizational diversity. On the other hand, if the level of conflict is low and diversity is high, innovation performance is high even if the level of destructive leadership is high. In this case, it was found that even if destructive leadership exists, the conflict between team members is low, so it shows stronger team cohesion and cooperation. Therefore, it was inferred that the innovation process and performance can be influenced according to the conditions of conflict and the level of organizational diversity. Thus, our findings highlight new insights to understand the impact of conflict and organizational diversity dynamics on team innovation performance along with disruptive leadership. In sum, this study has contributed to uncovering the factors that can maximize the negative effect of destructive leadership on team innovation performance. Despite several limitations, our research on substantial moderators provides useful insights for firms that wish to manage the negative work environment and the key condition which drive teams’ innovation.

## Data availability statement

The raw data supporting the conclusions of this article will be made available by the authors, without undue reservation, to any qualified researcher.

## Author contributions

SBC was the principal researcher and prepared the first draft of the article and added valuable theoretical and methodological insights based on his knowledge and expertise regarding the topic. S-WK supervised the study and refined the draft into a publishable article. KBJ collected the data and performed empirical analysis, and in addition to motivating the publication of this article. All authors have read and agreed to the submitted version of the manuscript.

## Appendix A

**Destructive Leadership (α = 0.879)** (Mitchell and Ambrose, 2007)

1. My team leader ridicules me.
2. My team leader tells me my thoughts or feelings are stupid.
3. My team leader puts me down in front of others.
4. My team leader makes negative comments about me to others.
5. My team leader tells me I’m incompetent.

**Intra-team Conflict (α = 0.904)** (Jehn, 1995)

1. Our team tends to have personality conflicts between members.
2. Our team tends to have emotional conflicts between members.
3. Our team tends to have personality conflict between members.
4. Our team tends to have tension between the members.

**Team Innovation Performance (α = 0.877)** (Pirola-Merlo and Mann, 2004)

1. My team’s recent output is new.
2. My team’s recent output is creative.
3. My team’s recent output is useful.
4. My team’s recent output is innovative.
